# Understanding Teach-Back and Teach-To-Goal Strategies Embedded in Support Calls for a Health Literacy-Sensitive Childhood Obesity Treatment Trial

**DOI:** 10.3928/24748307-20210713-01

**Published:** 2021-07

**Authors:** Maryam Yuhas, Jamie Zoellner, Xiaolu Hou, Ramine Alexander, Jennie Hill, Wen You, Paul Estabrooks

## Abstract

**Background:**

Low caregiver health literacy (HL) is related to increased obesity risk for their children. Teach-Back and Teach-to-Goal (TB/TTG) are strategies that may improve comprehension of key concepts for people who have low HL but have yet to be examined in the context of childhood obesity treatment.

**Objective:**

This study evaluated TB/TTG strategies integrated within support calls delivered to caregivers as part of a 3-month childhood obesity intervention.

**Methods:**

Ninety-four caregivers (60% Black, 42% high school education or less, 53% with income ≤$29,999, and 34% low HL) with overweight/obese children age 8 to 12 years enrolled in a childhood obesity intervention. Caregiver HL was assessed at baseline using the Newest Vital Sign and caregivers were dichotomized to low and adequate HL groups for analyses. Caregivers received 6 bi-weekly support calls that alternated with in-person, family sessions. Call completion rates, comprehension of key content (correct responses on TB/TTG questions), and satisfaction with support calls were evaluated. Qualitative information on call satisfaction was gathered at the 3-month time point.

**Key Results:**

Average completion rate across all calls was 62% with a mean call time of 26 minutes (no significant difference between HL groups). Caregivers had an average score of 0.90 out of 1 when evaluating overall call comprehension by scoring TB/TTG performance. Content comprehension in calls 1, 3, and 4 was significantly higher among caregivers with adequate HL relative to low HL (*p* < .1). Caregivers from both HL groups felt satisfied with calls [9.1 (2.0)/10-point scale] and agreed that calls helped them learn class material better [8.1 (2.7)]. Qualitatively, caregivers provided 81 (75%) positive responses (e.g., good content) and 27 (25%) negative responses (e.g., too lengthy) regarding the support calls.

**Conclusions:**

Support calls using TB/TTG strategies were feasible, well received, and should be considered for incorporation into childhood obesity interventions. **[*HLRP: Health Literacy Research and Practice*. 2021;5(3):e208–e217.]**

**Plain Language Summary:**

This study evaluated support calls that used Teach-Back and Teach-to-Goal health literacy strategies as part of a childhood obesity treatment trial. Support calls were well accepted and facilitated comprehension of the key learning objectives in caregivers, regardless of health literacy level. These strategies should be considered for incorporation into childhood obesity treatment interventions to increase uptake of main concepts.

Prevalence of childhood obesity has risen dramatically over the past 18 years, especially in racial/ethnic minority and low-income communities (Fryar et al., 2018; Williams et al., 2018). Although there are many factors related to higher rates of obesity in racial/ethnic minority and low-income populations, health literacy (HL) may be one contributing factor. Low HL (LHL) has been found to be more prevalent among racial/ethnic minority, rural, and low socioeconomic populations (Christy et al., 2017; Zahnd et al., 2009). Specifically related to parents/caregivers, studies have linked low parent/caregiver HL to limited knowledge and comprehension of child health information, reduced engagement in family health behaviors, and poor child health outcomes (DeWalt & Hink, 2009; Sanders et al., 2009). Furthermore, the current body of parent and caregiver studies suggests that interventions applying HL strategies have a positive impact on health-related knowledge, behaviors, and outcomes (DeWalt & Hink, 2009; Herman et al., 2012; Zoellner et al., 2017).

There are several strategies to improve written and verbal communications for individuals with LHL. These include enhancing the readability of written materials, limiting the number of concepts introduced at one time, and supporting textual instructions with pictures and/or video (Morrison et al., 2019). Verbal communication strategies include the Teach-Back (TB) method that has been endorsed by the American Medical Association (Huizinga et al., 2008; Weiss, 2007). To use this strategy, health care professionals ask participants to repeat instructions or explain key concepts using their own words. TB can be performed to assess participant comprehension as well as to clarify and reinforce key messages. TB is often paired with the Teach-to-Goal (TTG) approach, which includes multiple rounds of TB until participants achieve their learning goals (DeWalt et al., 2009).

TB and TTG (TB/TTG) strategies targeting caregivers can promote a variety of beneficial child health behaviors, including reducing medication dosing errors and improving medication adherence (DeWalt et al., 2012; Negarandeh et al., 2013; Wilson et al., 2012). Several behavioral intervention studies have incorporated TB/TTG strategies into telephone support calls (Goessl et al., 2019; Porter et al., 2016) including one intervention aimed at parents of infants to prevent childhood obesity (Sanders et al., 2014). However, there has been no research on using this approach in the context of childhood obesity treatment.

The objective of this study was to understand how caregivers participating in the HL-sensitive childhood obesity treatment trial used TB/TTG embedded within support calls and to explore if there were differences in how caregivers perceived or benefited from the process by caregiver HL status. It was hypothesized that the use of TB/TTG could be beneficial but could also be perceived as repetitive by caregivers. Finally, the childhood obesity treatment intervention was designed to transition from research implementation to sustained community delivery, which included an ancillary objective to examine potential implementation differences of the support calls by delivery staff type.

## Methods

### Study Design and Intervention Structure

This study is a secondary analysis of a large, three-wave pilot community-based childhood obesity treatment trial, iChoose, conducted in 2014–2015. A complete description of the iChoose program and outcomes can be found elsewhere (Hill et al., 2019; Hou, 2017). In brief, iChoose is a family-based childhood obesity treatment program that was designed using a HL universal precautions approach (i.e., creating materials with the assumption that all participants were had risk factors for not understanding material; Zoellner et al., 2017). A pilot trial was conducted to explore the potential reach, effectiveness, adherence, and implementation feasibility of the program. The 3-month iChoose program included (1) bi-weekly family nutrition and exercise sessions; (2) bi-weekly caregiver telephone support calls to set goals, resolve barriers, and reinforce content using TB/TTG strategies between family nutrition and exercise sessions; (3) twice-weekly exercise sessions; (4) workbooks for both caregivers and children; and (5) children's newsletters to reinforce content.

**Figure 1.**
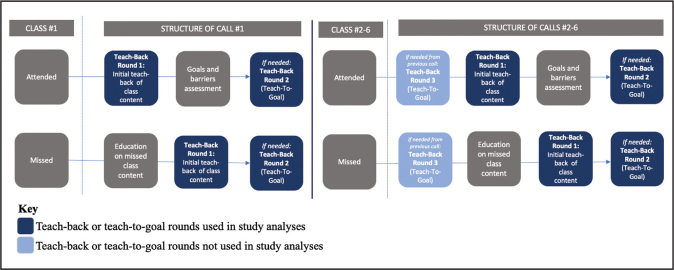
Support call flow and structure.

Caregivers received the support calls 4 to 12 days after each family session, regardless of attendance. Whenever a caregiver missed the preceding family session, a modified version of the call script was used that included a brief verbal lesson. To guide the telephone support calls, six structured call scripts were developed to correspond to each family session. Each call included 4 to 5 TB questions per script to evaluate and promote comprehension as well as support retention of key learning objectives. A summary of topics covered within each call and example TB questions can be found in **Table 1**. Any caregiver who answered a TB question incorrectly was given additional instruction and offered two additional chances to answer the question correctly, referred to as TTG (up to 2 rounds during the same call and, if needed, a third round during the next call; see **Figure 1**). The Virginia Tech Institutional Review Board approved this study, and all caregivers provided informed consent. Caregivers received $25, $25, and $50 gift cards at the baseline, 3-month, and 6-month health screenings, respectively.

**Table 1:**
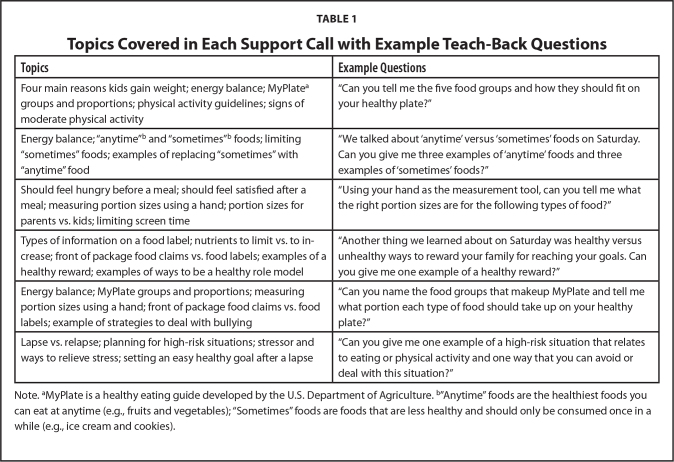
Topics Covered in Each Support Call with Example Teach-Back Questions

**Topics**	**Example Questions**
Four main reasons kids gain weight; energy balance; MyPlate^a^ groups and proportions; physical activity guidelines; signs of moderate physical activity	“Can you tell me the five food groups and how they should fit on your healthy plate?”
Energy balance; “anytime”^b^ and “sometimes”^b^ foods; limiting “sometimes” foods; examples of replacing “sometimes” with “anytime” food	“We talked about ‘anytime’ versus ‘sometimes’ foods on Saturday. Can you give me three examples of ‘anytime’ foods and three examples of ‘sometimes’ foods?”
Should feel hungry before a meal; should feel satisfied after a meal; measuring portion sizes using a hand; portion sizes for parents vs. kids; limiting screen time	“Using your hand as the measurement tool, can you tell me what the right portion sizes are for the following types of food?”
Types of information on a food label; nutrients to limit vs. to increase; front of package food claims vs. food labels; examples of a healthy reward; examples of ways to be a healthy role model	“Another thing we learned about on Saturday was healthy versus unhealthy ways to reward your family for reaching your goals. Can you give me one example of a healthy reward?”
Energy balance; MyPlate groups and proportions; measuring portion sizes using a hand; front of package food claims vs. food labels; example of strategies to deal with bullying	“Can you name the food groups that makeup MyPlate and tell me what portion each type of food should take up on your healthy plate?”
Lapse vs. relapse; planning for high-risk situations; stressor and ways to relieve stress; setting an easy healthy goal after a lapse	“Can you give me one example of a high-risk situation that relates to eating or physical activity and one way that you can avoid or deal with this situation?”

Note.

a MyPlate is a healthy eating guide developed by the U.S. Department of Agriculture.

b ”Anytime” foods are the healthiest foods you can eat at anytime (e.g., fruits and vegetables); “Sometimes” foods are foods that are less healthy and should only be consumed once in a while (e.g., ice cream and cookies).

### Target Population, Eligibility, and Recruitment

The iChoose pilot trial recruited families in the Dan River Region of Virginia, a federally designated, medically underserved area (Health Resources & Services Administration, n.d.). According to U.S. Census data, approximately 34% of residents in the region are Black, 15% of the residents live below the poverty line, and only 19% have obtained a bachelor's degree or higher (U.S. Census Bureau, 2019). To be eligible to participate, caregiver and child dyads needed to be English-speaking and have no contraindications for physical activity. Children needed to be age 8 to 12 years, with a body mass index (BMI) at or greater than the 85th percentile for their age and sex. Families were excluded if children had major cognitive impairments or if they were participating in another childhood obesity treatment program.

## Measures and Data Collection

### Demographics

Caregiver demographic information was collected using verbally administered questions including age, race/ethnicity, gender, annual household income, and caregiver education level. Caregiver HL was assessed using the validated Newest Vital Sign (NVS), a screening tool consisting of six verbally administered questions to evaluate one's ability to read and apply information from a nutrition label (Weiss et al., 2005). Established cut-points were used to categorize caregivers as having low (LHL, 0–3) or adequate HL (AHL, 4–6).

### Support Call Process Data

The iChoose program was pilot tested using a three-wave implementation process that progressively increased the role of community staff in delivery. Trained research partners (*n* = 2) delivered all support calls in Wave 1, whereas call delivery responsibilities were divided between community (*n* = 5) and research partners (*n* = 2) in Waves 2 and 3. For each support call, delivery staff recorded call attempts, call completion status, and call length. Support call completion was assessed as the proportion of the six possible support calls completed by the caregiver. The average call duration was calculated as the mean number of minutes of all completed support calls.

### Mean Call Performance: Teach-Back and Teach-To-Goal Outcome Scores

Performance on TB/TTG questions within support calls was evaluated using data recorded during support calls. Using guidance from previous literature (Goessl et al., 2019; Porter et al., 2016), three separate measures were created to capture the different dimensions of call performance. First, the average number of TB rounds needed (either 1 or 2) was calculated by averaging rounds needed across calls. For this variable, a higher score represented a lower call performance. Second, the proportion of correct questions on Round 1 was calculated by summing the number of questions answered correctly during the first round and dividing by the number of questions asked during each call. Third, a comprehensive score proportion variable was calculated using reverse scoring procedures as described by Goessl et al. (2019). Specifically, questions were first individually scored so that 2 = correct on the first round, 1 = correct on the second round, and 0 = incorrect on the second round. The scores were summed across each call, so that the highest score represents that all questions were answered correctly on the first round. Scores were then converted into proportions by dividing by the highest possible points per call. The comprehensive score proportion variable focused exclusively on the first 2 rounds of TB/TTG even though a third round of TB/TTG was given to caregivers who needed it. This approach was used to reduce missing data resulting from caregivers who did not complete a third round of TB/TTG when needed.

### Summative Evaluation Feedback

Caregiver perceptions of the support calls were assessed using a verbally administered summative evaluation at the end of the 3-month program. Trained graduate research assistants interviewed caregivers using structured qualitative questions and recorded their responses. The questions asked caregivers to identify barriers to call completion, ways the delivery staff might have assisted to promote call completion, and overall likes and dislikes about the calls. Eleven quantitative questions assessed overall satisfaction with the calls on 10-point Likert-type scales (1 = *strongly disagree* or *completely dissatisfied*, 10 = *strongly agree* or *completely satisfied*).

### Support Call Implementation Data

The total amount of time needed to implement support calls was estimated using data on call lengths for completed calls and the number of unsuccessful call attempts made according to protocol (i.e., 2 minutes added for each unsuccessful call attempt). Fidelity in implementing the support calls was assessed based on a checklist of key components in the guided scripts that were completed by implementation staff during each call. These scores were converted into percentages for each call, then averaged across the six calls for both community and research partners.

## Data Analysis

All statistical tests were completed using SPSS version 26.0 and Microsoft Excel. Descriptive statistics were computed to characterize demographic variables and summarize other data. One-way ANOVAs (analysis of variance) tests were used to examine differences between HL level by support call completion, length, comprehension, and satisfaction. Because of the exploratory nature of this trial the probability value to indicate significance was set *a priori* at *p* < .1 for all quantitative comparisons. For qualitative data, a manifest content analysis approach was used (Bengtsson, 2016). Two researchers independently reviewed responses to the structured questions, coded for common themes, and then met to resolve discrepancies.

## Results

A total of 94 caregivers participated in the iChoose pilot trial. Caregivers were predominantly female (94%), 42% had a high school education or less, and 53% had an income below $29,999. In addition, participating caregivers were mostly Black (60%) or White (38%). The average age of caregivers in the sample was 40 ± 8.8 years, and nearly one-half were married (49%). Of enrolled caregivers, 32 (34%) were categorized as having LHL and 62 (66%) were categorized as having AHL. The average completion rate across all six biweekly calls was 62% (range, 50%–74%) and did not vary significantly by LHL (58 ± 41%) versus AHL (64 ± 39%) status. Completed support calls took an average of 26.3 ± 8.3 minutes and did not vary significantly by LHL (27.9 ± 10.1 minutes) versus AHL (25.5 ± 7.4 minutes) status.

Significant differences in call comprehension were found for calls 1, 3 and 4, where caregivers with AHL performed better (**Table 2**). For calls 1 and 4, a significant difference was observed by caregiver HL status, in the number of TB rounds needed, proportion of round 1 questions correct, and the comprehensive score (*p* < .1). For call 3, a significant difference was observed by caregiver HL status, in the proportion of round 1 questions correct and comprehensive score proportion. Across the six calls, the average number of TB rounds needed ranged from 1.04 to 1.33, with an average score of 1.13 (*SD* [standard deviation] = 0.12) from a possible of 2 total rounds. TB questions were answered correctly on the first round 85% of the time. When evaluating overall comprehensive scores, caregivers had an average proportional score of 0.90 (*SD* = 0.11) out of 1. When averaged across all calls, and compared to caregivers with LHL, caregivers with AHL required significantly fewer TB rounds, had a higher proportion of TB questions answered correctly on the first round, and had higher comprehensive scores.

**Table 2:**
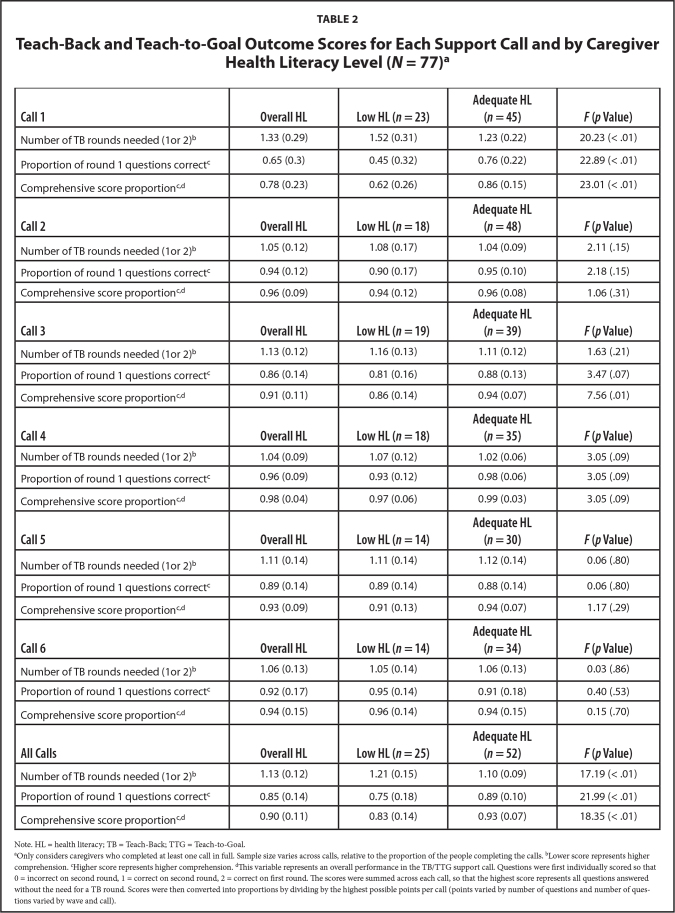
Teach-Back and Teach-to-Goal Outcome Scores for Each Support Call and by Caregiver Health Literacy Level (*N* = 77)^a^

**Call 1**		**Overall HL**	**Low HL (*n* = 23)**	**Adequate HL (*n* = 45)**	***F* (*p* Value)**
	Number of TB rounds needed (1or 2)^b^	1.33 (0.29)	1.52 (0.31)	1.23 (0.22)	20.23 (< .01)
	Proportion of round 1 questions correct^c^	0.65 (0.3)	0.45 (0.32)	0.76 (0.22)	22.89 (< .01)
	Comprehensive score proportion^c,d^	0.78 (0.23)	0.62 (0.26)	0.86 (0.15)	23.01 (< .01)
**Call 2**		**Overall HL**	**Low HL (*n* = 18)**	**Adequate HL (*n* = 48)**	***F* (*p* Value)**
	Number of TB rounds needed (1or 2)^b^	1.05 (0.12)	1.08 (0.17)	1.04 (0.09)	2.11 (.15)
	Proportion of round 1 questions correct^c^	0.94 (0.12)	0.90 (0.17)	0.95 (0.10)	2.18 (.15)
	Comprehensive score proportion^c,d^	0.96 (0.09)	0.94 (0.12)	0.96 (0.08)	1.06 (.31)
**Call 3**		**Overall HL**	**Low HL (*n* = 19)**	**Adequate HL (*n* = 39)**	***F* (*p* Value)**
	Number of TB rounds needed (1or 2)^b^	1.13 (0.12)	1.16 (0.13)	1.11 (0.12)	1.63 (.21)
	Proportion of round 1 questions correct^c^	0.86 (0.14)	0.81 (0.16)	0.88 (0.13)	3.47 (.07)
	Comprehensive score proportion^c,d^	0.91 (0.11)	0.86 (0.14)	0.94 (0.07)	7.56 (.01)
**Call 4**		**Overall HL**	**Low HL (*n* = 18)**	**Adequate HL (*n* = 35)**	***F* (*p* Value)**
	Number of TB rounds needed (1or 2)^b^	1.04 (0.09)	1.07 (0.12)	1.02 (0.06)	3.05 (.09)
	Proportion of round 1 questions correct^c^	0.96 (0.09)	0.93 (0.12)	0.98 (0.06)	3.05 (.09)
	Comprehensive score proportion^c,d^	0.98 (0.04)	0.97 (0.06)	0.99 (0.03)	3.05 (.09)
**Call 5**		**Overall HL**	**Low HL (*n* = 14)**	**Adequate HL (*n* = 30)**	***F* (*p* Value)**
	Number of TB rounds needed (1or 2)^b^	1.11 (0.14)	1.11 (0.14)	1.12 (0.14)	0.06 (.80)
	Proportion of round 1 questions correct^c^	0.89 (0.14)	0.89 (0.14)	0.88 (0.14)	0.06 (.80)
	Comprehensive score proportion^c,d^	0.93 (0.09)	0.91 (0.13)	0.94 (0.07)	1.17 (.29)
**Call 6**		**Overall HL**	**Low HL (*n* = 14)**	**Adequate HL (*n* = 34)**	***F* (*p* Value)**
	Number of TB rounds needed (1or 2)^b^	1.06 (0.13)	1.05 (0.14)	1.06 (0.13)	0.03 (.86)
	Proportion of round 1 questions correct^c^	0.92 (0.17)	0.95 (0.14)	0.91 (0.18)	0.40 (.53)
	Comprehensive score proportion^c,d^	0.94 (0.15)	0.96 (0.14)	0.94 (0.15)	0.15 (.70)
**All Calls**		**Overall HL**	**Low HL (*n* = 25)**	**Adequate HL (*n* = 52)**	***F* (*p* Value)**
	Number of TB rounds needed (1or 2)^b^	1.13 (0.12)	1.21 (0.15)	1.10 (0.09)	17.19 (< .01)
	Proportion of round 1 questions correct^c^	0.85 (0.14)	0.75 (0.18)	0.89 (0.10)	21.99 (< .01)
	Comprehensive score proportion^c,d^	0.90 (0.11)	0.83 (0.14)	0.93 (0.07)	18.35 (< .01)

Note. HL = health literacy; TB = Teach-Back; TTG = Teach-to-Goal.

a Only considers caregivers who completed at least one call in full. Sample size varies across calls, relative to the proportion of the people completing the calls.

b Lower score represents higher comprehension.

c Higher score represents higher comprehension.

d This variable represents an overall performance in the TB/TTG support call. Questions were first individually scored so that 0 = incorrect on second round, 1 = correct on second round, 2 = correct on first round. The scores were summed across each call, so that the highest score represents all questions answered without the need for a TB round. Scores were then converted into proportions by dividing by the highest possible points per call (points varied by number of questions and number of questions varied by wave and call).

The summative evaluation was completed by 65 (69%) caregivers. Results indicated high levels of satisfaction and acceptability toward support calls, and ratings were similar for caregivers across HL status (**Table 3**). The one exception was caregivers with LHL expressed significantly (*p* = .056) stronger agreement that follow-up support calls helped improve family eating habits (9.3; *SD* = 1.9) compared to those with AHL (7.7; *SD* = 2.7). When asked about likes and dislikes regarding support calls, caregivers provided a total of 81 (75%) positive responses and 27 (25%) negative responses. **Table 4** summarizes the most common responses and caregiver examples. Descriptively, differences between HL level of caregivers emerged regarding the scheduling and format of the calls. Caregivers with AHL were more likely to report they had scheduling difficulties, for example, due to their busy schedule or being a single caregiver. Caregivers with LHL were more likely to report the format of the calls (e.g., too lengthy) made it hard for them to complete the calls.

**Table 3:**
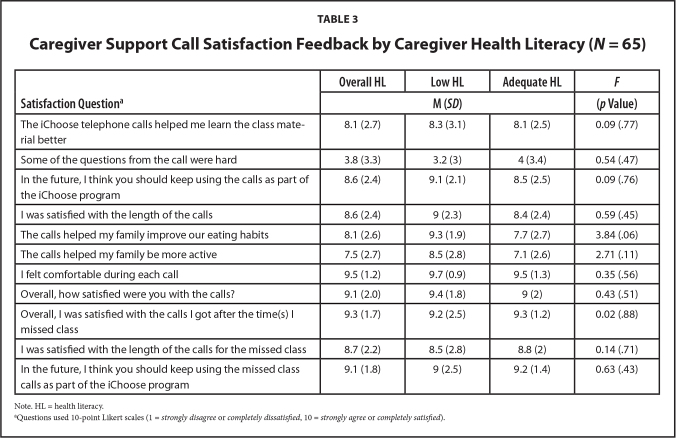
Caregiver Support Call Satisfaction Feedback by Caregiver Health Literacy (*N* = 65)

**Satisfaction Question^a^**	**Overall HL**	**Low HL**	**Adequate HL**	***F***
	**M (*SD*)**			**(*p* Value)**
The iChoose telephone calls helped me learn the class material better	8.1 (2.7)	8.3 (3.1)	8.1 (2.5)	0.09 (.77)
Some of the questions from the call were hard	3.8 (3.3)	3.2 (3)	4 (3.4)	0.54 (.47)
In the future, I think you should keep using the calls as part of the iChoose program	8.6 (2.4)	9.1 (2.1)	8.5 (2.5)	0.09 (.76)
I was satisfied with the length of the calls	8.6 (2.4)	9 (2.3)	8.4 (2.4)	0.59 (.45)
The calls helped my family improve our eating habits	8.1 (2.6)	9.3 (1.9)	7.7 (2.7)	3.84 (.06)
The calls helped my family be more active	7.5 (2.7)	8.5 (2.8)	7.1 (2.6)	2.71 (.11)
I felt comfortable during each call	9.5 (1.2)	9.7 (0.9)	9.5 (1.3)	0.35 (.56)
Overall, how satisfied were you with the calls?	9.1 (2.0)	9.4 (1.8)	9 (2)	0.43 (.51)
Overall, I was satisfied with the calls I got after the time(s) I missed class	9.3 (1.7)	9.2 (2.5)	9.3 (1.2)	0.02 (.88)
I was satisfied with the length of the calls for the missed class	8.7 (2.2)	8.5 (2.8)	8.8 (2)	0.14 (.71)
In the future, I think you should keep using the missed class calls as part of the iChoose program	9.1 (1.8)	9 (2.5)	9.2 (1.4)	0.63 (.43)

Note. HL = health literacy.

a Questions used 10-point Likert scales (1 = *strongly disagree* or *completely dissatisfied*, 10 = *strongly agree* or *completely satisfied*).

**Table 4:**
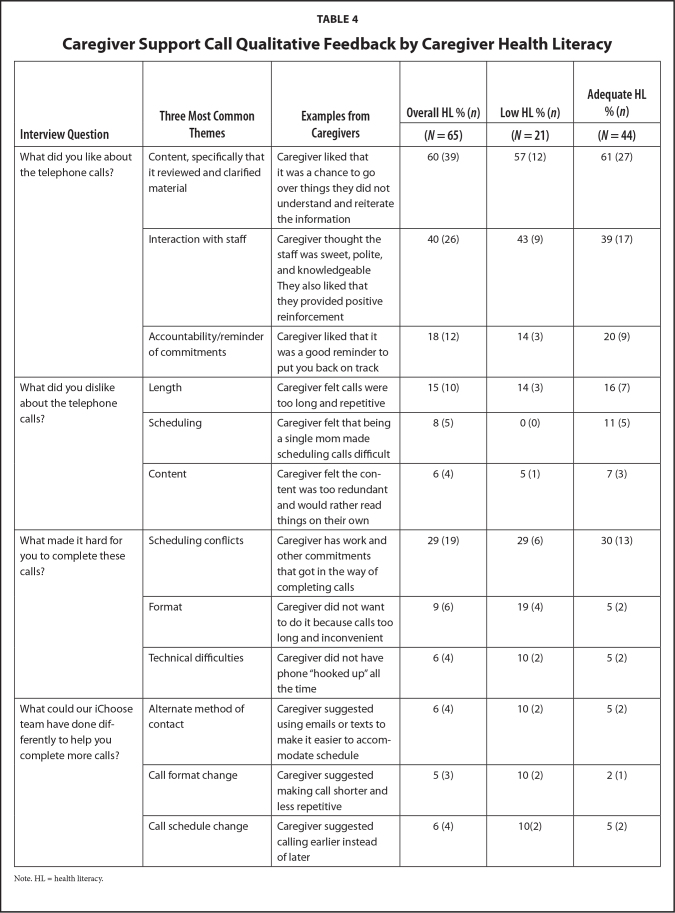
Caregiver Support Call Qualitative Feedback by Caregiver Health Literacy

**Interview Question**	**Three Most Common Themes**	**Examples from Caregivers**	**Overall HL % (*n*)**	**Low HL % (*n*)**	**Adequate HL % (*n*)**
			**(*N* = 65)**	**(*N* = 21)**	**(*N* = 44)**
What did you like about the telephone calls?	Content, specifically that it reviewed and clarified material	Caregiver liked that it was a chance to go over things they did not understand and reiterate the information	60 (39)	57 (12)	61 (27)
	Interaction with staff	Caregiver thought the staff was sweet, polite, and knowledgeable They also liked that they provided positive reinforcement	40 (26)	43 (9)	39 (17)
	Accountability/reminder of commitments	Caregiver liked that it was a good reminder to put you back on track	18 (12)	14 (3)	20 (9)
What did you dislike about the telephone calls?	Length	Caregiver felt calls were too long and repetitive	15 (10)	14 (3)	16 (7)
	Scheduling	Caregiver felt that being a single mom made scheduling calls difficult	8 (5)	0 (0)	11 (5)
	Content	Caregiver felt the content was too redundant and would rather read things on their own	6 (4)	5 (1)	7 (3)
What made it hard for you to complete these calls?	Scheduling conflicts	Caregiver has work and other commitments that got in the way of completing calls	29 (19)	29 (6)	30 (13)
	Format	Caregiver did not want to do it because calls too long and inconvenient	9 (6)	19 (4)	5 (2)
	Technical difficulties	Caregiver did not have phone “hooked up” all the time	6 (4)	10 (2)	5 (2)
What could our iChoose team have done differently to help you complete more calls?	Alternate method of contact	Caregiver suggested using emails or texts to make it easier to accommodate schedule	6 (4)	10 (2)	5 (2)
	Call format change	Caregiver suggested making call shorter and less repetitive	5 (3)	10 (2)	2 (1)
	Call schedule change	Caregiver suggested calling earlier instead of later	6 (4)	10(2)	5 (2)

Note. HL = health literacy.

Both research and community partners adhered to the guided call scripts with high fidelity (97% and 98%, respectively) with no significant difference between them (*p* = .392). Support calls delivered by researchers (27.2 ± 9.5 minutes) were not significantly longer on average than those delivered by community partners (25.4 ± 7.0 minutes)(*p* = .342).

## Discussion

This study provided novel information regarding the feasibility, acceptability, and implementation of support calls incorporating TB/TTG methods within a community-based childhood obesity treatment intervention. Support calls using TB/TTG strategies were well accepted and multiple rounds of TB appeared to have increased comprehension of the key learning objectives in caregivers with both AHL and LHL. The results of this study are consistent with research in other contexts that confirm the utility of TB/TTG strategies within support calls to improve uptake of key concepts. Two studies, one focusing on sugar-sweetened beverage consumption in adults and another in a diabetes prevention context, found that multiple rounds of TB/TTG were needed and improved comprehension of material for participants of varying HL levels, and overall, participants with AHL performed better (Goessl et al., 2019; Porter et al., 2016). In our study, the average number of TB rounds for both LHL and AHL were greater than 1, suggesting that even though AHL performed better, some caregivers in both HL groups benefited from an extra TB round. This finding, in combination with the existing literature, indicates the importance of utilizing TTG strategies as a universal precautions approach, to reinforce content for caregivers regardless of HL (Brega et al., 2015).

Because our pilot trial did not have a control group that received no TB/TTG calls, findings must be interpreted somewhat cautiously. Nonetheless, our study suggests that TB/TTG methods may be used iteratively to reinforce comprehension of program content that may not be well understood initially by caregivers with LHL compared to AHL. In this study, caregivers with LHL had significantly lower performance in calls 1, 3 and 4. Calls 1 and 3 both covered topics on portion sizes, and call 4 covered topics on food packaging and the nutrition label. These topics in the iChoose intervention may require additional reinforcement or adaptation. Porter et al. (2016) also found that participants with LHL had trouble understanding certain concepts within their support call that were related to questions that asked participants to apply numeracy skills. It is also worth noting that TB/TTG performance data can help to highlight not only the most challenging programming topics but also the most easily grasped ones. Collection and analysis of this type of data can serve an important role in future improvement and tailoring of intervention strategy so that valuable instructional resources can be used to target the most challenging concepts and individuals who may need the most support to understand them.

Another aspect of support call utility explored in this study was satisfaction of the support calls. Porter et al. (2016) also found overall positive perceptions of using support calls with TB/TTG strategies regardless of HL. These findings are not surprising given the evidence in the literature indicating that participants enjoy the individualized support, instruction, and follow-up provided by telephone calls incorporating TB (Zipp et al., 2010). Although a small number of caregivers in the iChoose program did express that the TB/TTG process was long and redundant, more seemed to appreciate the various forms of support offered by the calls. It is possible that the conversational non-text format used for these calls also helped caregivers feel comfortable during TB/TTG assessments.

There are also practical applications related to program implementation that can be derived from our study. First, some topics require additional attention based on the need for repeated rounds of TB/TTG. This could be due to inadequate coverage of a topic during face-to-face sessions or that some concepts are harder to retain over time. Regardless, this information can help program implementers target areas that may require more attention. Similarly, some content areas are more easily understood by participants as indicated by having no need to repeat TB questions. This information can be used to streamline follow-up calls to focus on more challenging content. Additional testing is needed to further explore the potential of TB/TTG strategies not only to clarify concepts following instruction but also to improve the quality of the original educational session.

Second, this trial was designed to transition program implementation from research staff to community staff to help support local sustainability. Importantly, the high level of fidelity demonstrated by the community-implementers was similar to the research-implementers. This suggests that support calls with embedded TB/TTG strategies can effectively be implemented by community organizations. However, based on feedback from community partners, support calls were also considered too resource intensive for community personnel to deliver without research staff support (Hill et al., 2019). Thus, when transitioning support calls using TB/TTG to practice, there should be importance placed on using strategies that can make these support calls less burdensome on community and clinical staff. For example, in a research trial that has followed this project, the live phone calls were converted to an Interactive Voice Response (IVR) automated format (Zoellner et al., 2019). Future research that compares the live support calls to the IVR automated support calls to understand if there are differences in call comprehension, satisfaction, utilization rates, and cost could be beneficial in moving TB/TTG strategies into community programs.

## Study Limitations

Due to location-specific adaptations, these findings may have limited generalizability to other communities, especially those that are more urban and/or that differ significantly from the Dan River Region demographically. On the other hand, because the Dan River Region is a federally designated medically under-served area known to experience educational, economic, and health disparities, similar regions for which these findings may be more generalizable are also likely to be some of the most in need of effective health promotion strategies. Also, this pilot trial did not have a control group. Finally, because this pilot study was not specifically powered to detect differences between care-giver HL status, the null finding for some outcomes and the exploratory *a priori* significance threshold of *p* < .1 should be considered when interpreting findings.

## Conclusions

In summary, our study demonstrated that it is acceptable and feasible to implement HL-sensitive TB/TTG strategies with high fidelity as part of a rural community-based childhood obesity intervention such that they are highly acceptable to caregivers. Support calls using TB/TTG should be considered as a strategy within childhood obesity interventions as a universal way to reinforce content, regardless of caregivers' HL level. Our findings also inform different possibilities to use these strategies while using limited resources more efficiently. Future studies should use randomized controlled trials that are specifically powered to detect differences between HL status to understand how call comprehension was associated with behavior change. Furthermore, future research should explore the feasibility and acceptability of more time and cost-efficient means of deploying these strategies (e.g., automated calls or messages) as well as moving beyond feasibility and acceptability to connect these strategies to primary outcomes of interest (e.g., child BMI z-scores, caregiver BMI).
